# Enhanced viral-mediated cochlear gene delivery in adult mice by combining canal fenestration with round window membrane inoculation

**DOI:** 10.1038/s41598-018-21233-z

**Published:** 2018-02-14

**Authors:** Hidekane Yoshimura, Seiji B. Shibata, Paul T. Ranum, Richard J. H. Smith

**Affiliations:** 1 Molecular Otolaryngology and Renal Research Laboratories, Carver College of Medicine, University of Iowa, Iowa City, IA 52242 USA; 2 Department of Otorhinolaryngology, Shinshu University School of Medicine, Matsumoto, Nagano, 390-8621 Japan; 3 Department of Otolaryngology - Head and Neck Surgery, Carver College of Medicine, University of Iowa, Iowa City, IA 52242 USA; 4 Interdisciplinary Graduate Program in Molecular & Cellular Biology, The University of Iowa Graduate College, University of Iowa, Iowa City, IA 52242 USA; 5 Iowa Institute of Human Genetics, Carver College of Medicine, University of Iowa, Iowa City, IA 52242 USA

## Abstract

Cochlear gene therapy holds promise for the treatment of genetic deafness. Assessing its impact in adult murine models of hearing loss, however, has been hampered by technical challenges that have made it difficult to establish a robust method to deliver transgenes to the mature murine inner ear. Here in we demonstrate the feasibility of a combined round window membrane injection and semi-circular canal fenestration technique in the adult cochlea. Injection of both AAV2/9 and AAV2/Anc80L65 via this approach in P15–16 and P56–60 mice permits robust eGFP transduction of virtually all inner hair cells throughout the cochlea with variable transduction of vestibular hair cells. Auditory thresholds are not compromised. Transduction rate and cell tropism is primarily influenced by viral titer and AAV serotype but not age at injection. This approach is safe, versatile and efficient. Its use will facilitate studies using cochlear gene therapy in murine models of hearing loss over a wide range of time points.

## Introduction

Current clinical treatment options for hereditary hearing loss are limited to hearing aids and cochlear implantation^1^. Although these interventions are effective, molecular therapies to preserve or restore biological hearing are highly sought after. Several successful reports have emerged using intra-cochlear gene therapy to restore auditory and/or balance function in neonatal mouse models of genetic deafness^2–7^. While these reports are noteworthy, the neonatal mouse undergoes postnatal inner ear maturation – auditory function is not fully mature until two weeks after birth. In contrast, humans are born with mature inner ears^8^. This difference compromises the direct translation of murine results across species and highlights the need to develop gene therapy delivery strategies that target the mature murine ear^9^.

**Figure 1:**
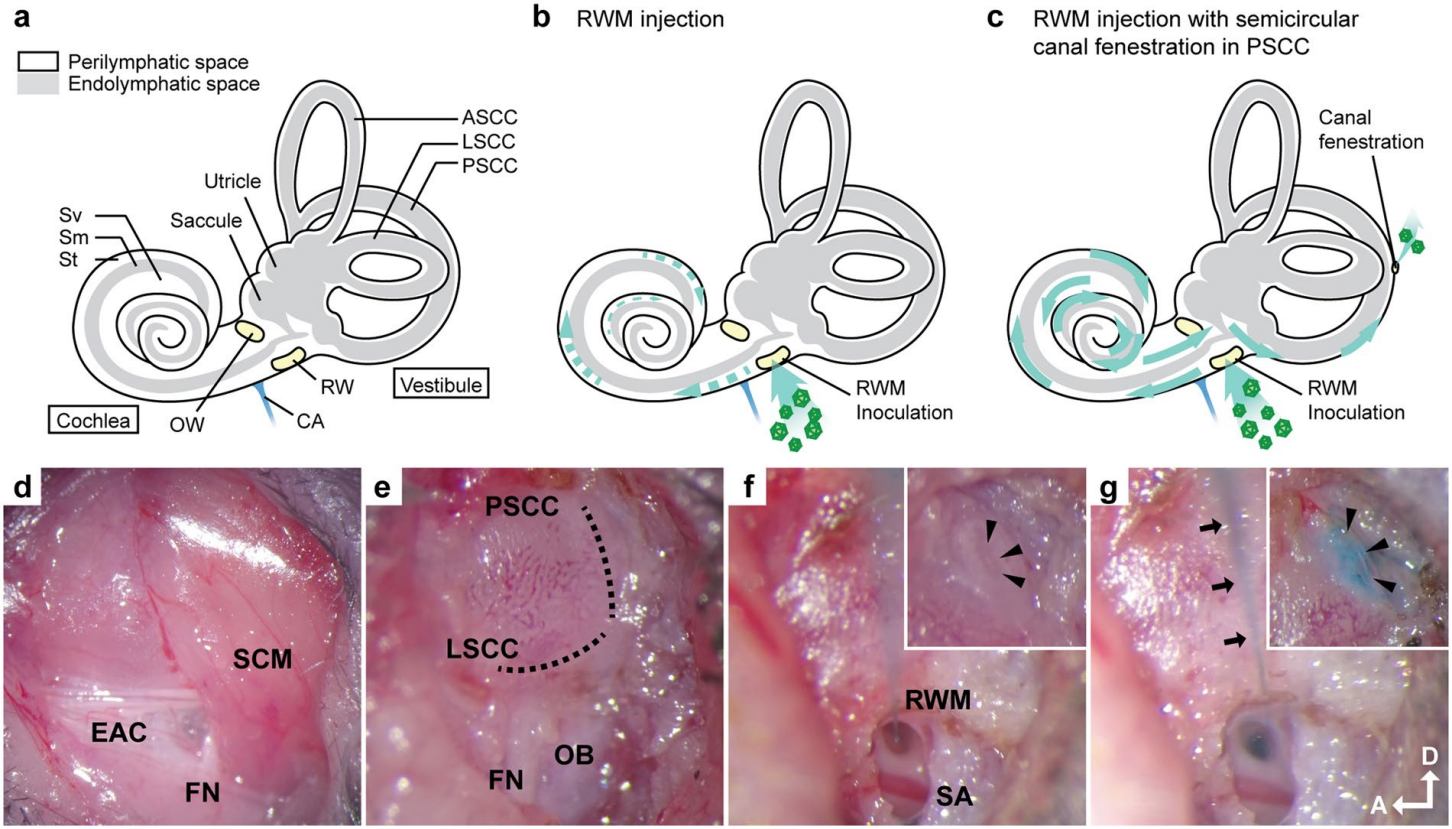
Inner ear schematic showing the RWM injection and the RWM + CF injection. Surgical approach for the RWM + CF injection. (**a**–**c**) Schematic diagram showing the anatomy of the inner ear (**a**), and the RWM (**b**) and RWM + CF (**c**) surgical approaches. Green dotted and solid lines indicate the predicted flow of injected vectors. Sv: scala vestibuli, Sm: scala media, St: scala tympani, OW: oval window, RW: round window, CA: cochlea aqueduct. (**d**–**g**) Surgical pictures showing the RWM + CF approach. Arrowheads indicate the hole in the PSCC before (**f**) and after (**g**) the inoculation. Arrows outline the glass pipette. EAC: external auditory canal, FN: facial nerve, SCM: sternocleidomastoid muscle, OB: otic bulla, SA: stapedial artery, D: dorsal, A: anterior.

A major obstacle to cochlear gene transfer in adult mice is the bony labyrinth, which precludes the safe delivery of a sufficient amount of therapeutic to the cochlear epithelium^10^ (Fig. 1a). The most common delivery technique is via the round window membrane (RWM)^11,12^. This approach is safe and clinically feasible, and is used for cochlear implantation in humans^13^. However, the bony labyrinth limits injection volume. In addition, the highly sensitive cochlear mechanosensory tissue is vulnerable to both the pressure and volume requirements associated with RWM injection techniques. As a result, hearing is often not preserved^14–16^, and only relatively low transgene expression is achieved, typically limited to the basal turn of the cochlea^17^. Chien and colleagues, for example, injected AAV8-CMV-eGFP through the RWM and showed that transduction efficiency of inner hair cells (IHCs) was 12–31% with apical to basal gradient. These mice also sustained a 30 dB auditory brainstem response (ABR) threshold shift, consistent with injection-associated cochlear damage^12^.

An ideal injection method would be nontraumatic and permit wide distribution of the therapeutic throughout the cochlear duct. Recently, Suzuki *et al*. showed that direct infusion of AAV2/Anc80L65 into the posterior semicircular canal (PSCC) in the cochlea of 6-week-old mice resulted in transduction of all IHCs throughout the spiral duct and most apical turn outer hair cells (OHCs), while preserving auditory function^16^. One limitation of this study was that it focused solely on the utilization of AAV2/Anc80L65; other serotypes were not screened. Of note, in neonatal mice injected with AAV8 via a PSCC canalostomy, transduction of IHCs was 69–76%^18^, suggesting that transduction efficiency with this approach may be serotype dependent. Additionally, one technical difficulty of the PSCC canalostomy injection is that it is impossible to determine whether the viral suspension is administered into the endolymphatic or perilymphatic space of PSCC^19,20^.

We sought to investigate a *trans*-RWM cochlear delivery method that enables high-efficiency apex-to-base transduction while preserving hearing. A drug delivery method that combines cochleostomy with canalostomy has been described in adult mice. This method showed a preferential perfusion flow pattern from the base to the apex in scala tympani that resulted from the creation of an ‘exit hole’ in the posterior semi-circular canal (PSCC). Drug concentration gradients were reduced and the drug distribution pattern was similar throughout all turns of the cochlea^21^. To our knowledge, a *trans*-RWM injection combined with canal fenestration (RWM + CF) has not been considered or evaluated as an approach for cochlear gene therapy.

Here, we assessed the following: (1) injection method (RWM injection - Fig. 1b versus RWM + CF injection - Fig. 1c) from the perspective of both transduction efficiency in IHCs and auditory function after the injection; (2) injection time point (postnatal day 15–16 [P15–16] versus P56–60); (3) injection titer (3.90 × 10^13^ vg/ml vs. 1.40 × 10^12^ vg/ml); (4) AAV serotype (AAV2/9 vs. AAV2/Anc80L65); and (5) off-target effects (transduction of the cerebellum and contralateral ear). We demonstrate that the RWM + CF approach is ideally suited for the mature murine inner ear. Both AAV serotypes efficiently transduce auditory cell types, with near complete transduction of IHCs in all turns of the cochlea without impacting auditory thresholds as measured by click and pure tone responses (8 kHz, 16 kHz, 24 kHz and 32 kHz). We show that transduction efficiency depends on the viral titer and AAV serotype, but importantly is independent of animal age at the time of surgery. Collectively our results demonstrate that the RWM + CF approach is a safe and efficient method to transduce IHCs in the adult murine inner ear. Its use will facilitate further studies using cochlear gene therapy in different murine models of hearing loss over a range of time points.

## Results

### RWM injection with AAV2/9: limited IHC transduction in basal turn with auditory threshold shift

**Figure 2:**
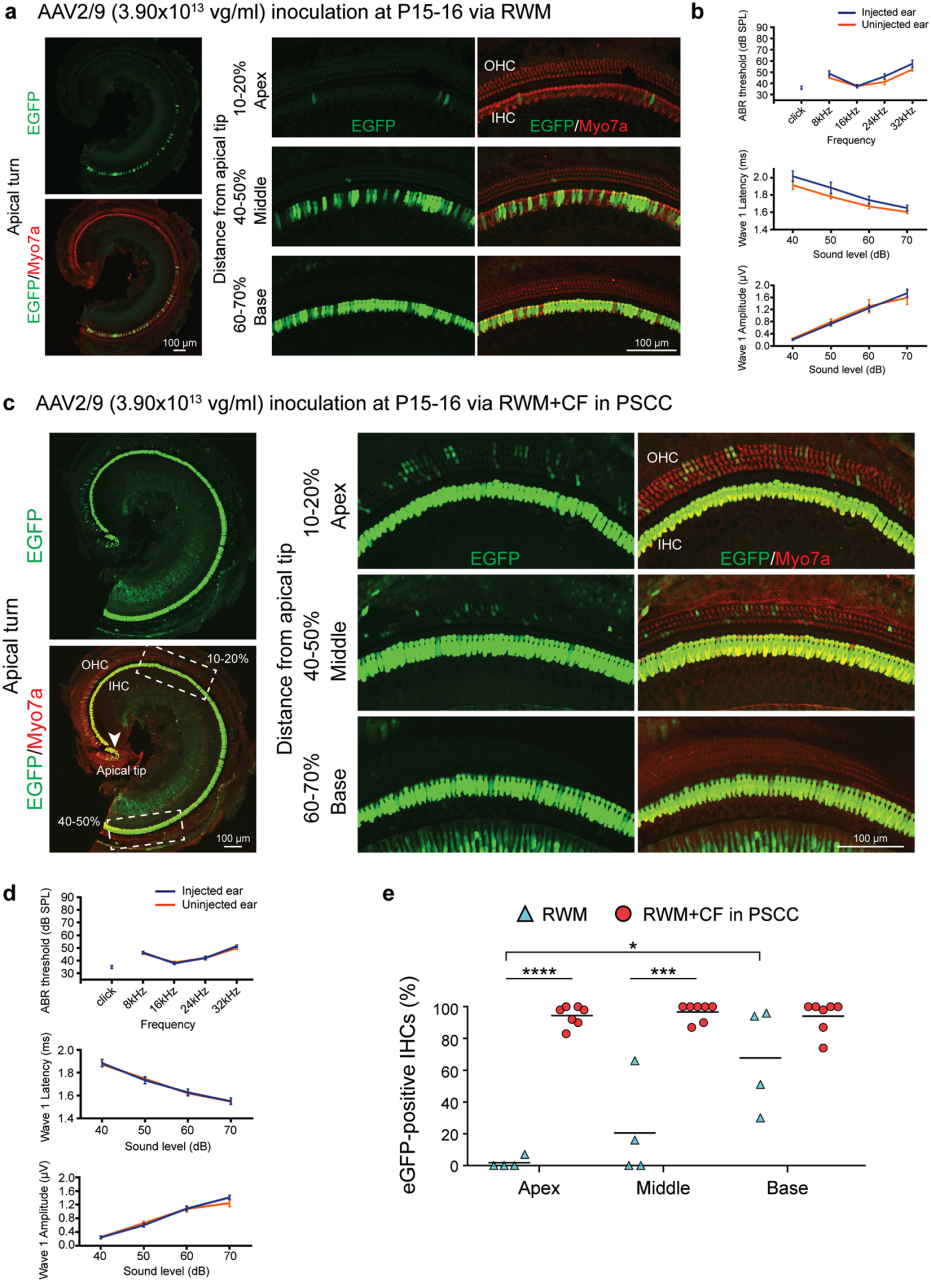
The RWM + CF approach improves cochlear transgene delivery as compared to the RWM approach. Cochleae were harvested 2 weeks after AAV2/9 (3.90 × 10^13^ vg/ml) inoculation (delivered at P15–16), stained with Myo7a (red) for labelling hair cells, and imaged for native eGFP (green). (**a**) Representative low magnification images of whole-mount apical turns and high magnification images of the apex, middle and base after the RWM injection. (**b**) Click and tone-burst ABR thresholds, P1 latencies and amplitudes in injected ears (n = 4) and uninjected contralateral ears (n = 4) 2 weeks after the RWM injection. Data are means ± SEM. (**c**) Representative low magnification images of whole-mount apical turns and high magnification images of the apex, middle and base after the RWM + CF injection. (**d**) Click and tone-burst ABR thresholds, P1 latencies and amplitudes in injected ears (n = 7) and uninjected contralateral ears (n = 7) 2 weeks after the RWM + CF injection. Data are means ± SEM. (**e**) Quantitative comparison of IHC transduction efficiency using AAV2/9 (3.90 × 10^13^ vg/ml) assessed in 400 µm segments across different regions of the cochlea (apex, middle and base) following either a RWM injection (n = 4) or a RWM + CF injection (n = 7) in P15–16 mice. Data are means; Student’s *t*-test and one-way ANOVA with Bonferroni’s correction: ****P < 0.0001, ***P < 0.001, *P < 0.05.

The transduction profile following RWM inoculation was investigated by quantitating cochlear eGFP expression in whole mount preparations of the membranous labyrinth 2 weeks after the P15–16 delivery of AAV2/9 (3.90 × 10^13^ vg/ml). We observed variable transduction efficiency across tonotopic positions in IHCs: apex 1.9%, middle 20.8% and base 68.1% (Fig. 2a and e). ABR and click-evoked Peak 1 (P1) latency responses 2 weeks after viral injection showed ABR threshold shifts of 5 dB at 24 kHz and 32 kHz as compared to uninjected controls; P1 latencies were slightly elevated (Fig. 2b and Supplementary Fig. S1).

### RWM + CF injection with AAV2/9: robust transgene expression without auditory threshold shift

The injection of AAV2/9 (3.90 × 10^13^ vg/ml) at P15–16 using the RWM + CF approach resulted in robust transduction of IHCs throughout the cochlea: apex 94.6%, middle 96.8% and base 94.2% (Fig. 2c and e). eGFP expression in OHCs was limited (Fig. 2c). Auditory thresholds and P1 latencies and amplitudes in injected and uninjected ears were comparable (Fig. 2d and Supplementary Fig. S1). These findings suggest that cochlear gene delivery using a RWM + CF approach facilitates high and uniform transduction of IHCs throughout the cochlea without altering auditory function.

### Transduction efficiency in IHCs: independent of injection time point

**Figure 3:**
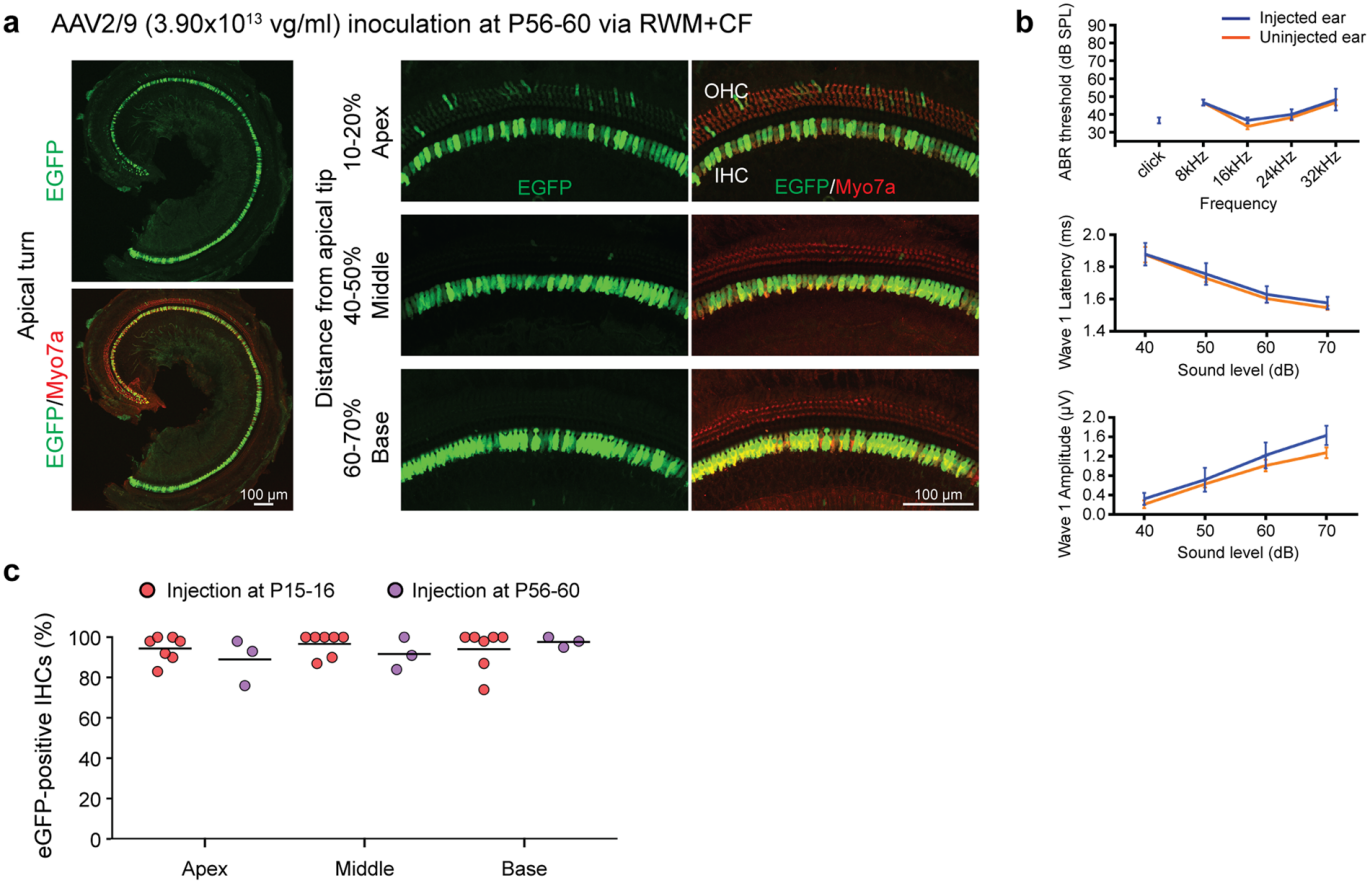
AAV2/9 delivery via the RWM + CF approach in P56–60 mice shows robust transduction of IHCs. (**a**) Representative low magnification images of whole-mount apical turns and high magnification images of the apex, middle and base 2 weeks after delivering AAV2/9 (3.90 × 10^13^ vg/ml) via a RWM + CF approach in P56–60 mice. Cochleae were stained with Myo7a (red) for labelling hair cells and imaged for native eGFP (green). (**b**) Click and tone-burst ABR thresholds, P1 latencies and amplitudes in injected ears (n = 3) and uninjected contralateral ears (n = 3) 2 weeks after the RWM + CF injection. Data are means ± SEM. (**c**) Quantitative comparison of IHC transduction efficiency using AAV2/9 (3.90 × 10^13^ vg/ml) assessed in 400 µm segments across different regions of the cochlea (apex, middle and base) following the RWM + CF injection of P15–16 or P56–60 mice. Data are means; Student’s *t*-test and one-way ANOVA with Bonferroni’s correction. ****P < 0.0001, ***P < 0.001, *P < 0.05.

To investigate the impact of age at time of injection, we treated P56–60 mice with AAV2/9 (3.90 × 10^13^ vg/ml). IHC transduction efficiency was 89.7% in the apical, 92.2% in the middle, and 98.1% in the basal turns of the cochlea; there were no injection-associated changes in auditory thresholds (Fig. 3a–c and Supplementary Fig. S1).

### Cochlear transduction: dose- and AAV serotype-dependent

**Figure 4:**
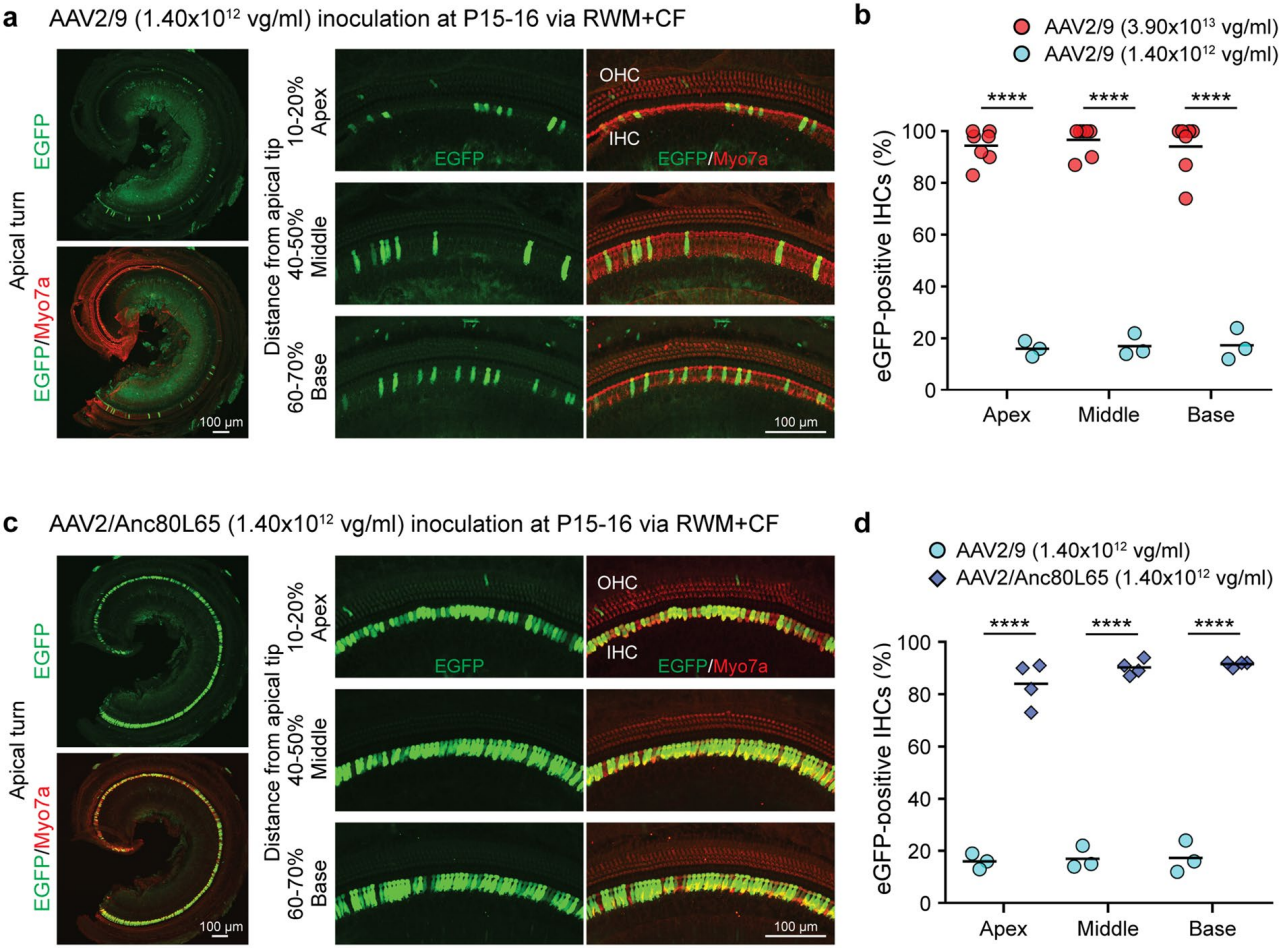
Dose- and serotype- dependency of AAV vector IHC transduction following the RWM + CF injection. Cochleae were harvested 2 weeks after the RWM + CF injection (delivered at P15–16), stained with Myo7a (red) for labelling hair cells, and imaged for native eGFP (green). Data are means; Student’s *t*-test and one-way ANOVA with Bonferroni’s correction. ****P < 0.0001. (**a**) Representative low magnification images of whole-mount apical turns and high magnification images of the apex, middle and base with AAV2/9 (1.40 × 10^12^ vg/ml; 1/28 of the high titer). (**b**) Quantitative comparison of IHC transduction efficiency using two different AAV2/9 titers (3.90 × 10^13^ vg/ml, n = 7 vs 1.40 × 10^12^ vg/ml, n = 3), assessed in 400 µm segments across different regions of the cochlea (apex, middle and base). (**c**) Representative low magnification images of whole-mount apical turns and high magnification images of the apex, middle and base with AAV2/Anc80L65 (1.40 × 10^12^ vg/ml). (**d**) Quantitative comparison of IHC transduction efficiency using equal doses of two serotypes (AAV2/9, n = 3 vs AAV2/Anc80L65, n = 4) assessed in 400 µm segments across different regions of the cochlea (apex, middle and base).

We used the RWM + CF approach to compare two AAV2/9 titers −3.90 × 10^13^ vg/ml and 1.40 × 10^12^ vg/ml – and found that at the lower titer (1/28 of the high titer), IHC transduction efficiency was significantly reduced: apex 16.7%, middle 17.4% and base 18.0% (Fig. 4a and b). We next compared transduction using two serotypes – AAV2/Anc80L65 and AAV2/9 – at 1.40 × 10^12^ vg/ml. With AAV2/Anc80L65, IHCs transduction efficiency was superior: apex 84.5%, middle 90.8% and base 91.9%. These results imply that transduction efficiency is both titer and AAV serotype dependent. AAV2/Anc80L65 demonstrated superior transduction to AAV2/9 when the titers were matched, however if higher titers of AAV2/9 were used, IHC transduction of the two serotypes was comparable (Fig. 4c and d).

### Vestibular organs: transduction occurs with either posterior or lateral semi-circular canal fenestration

**Figure 5:**
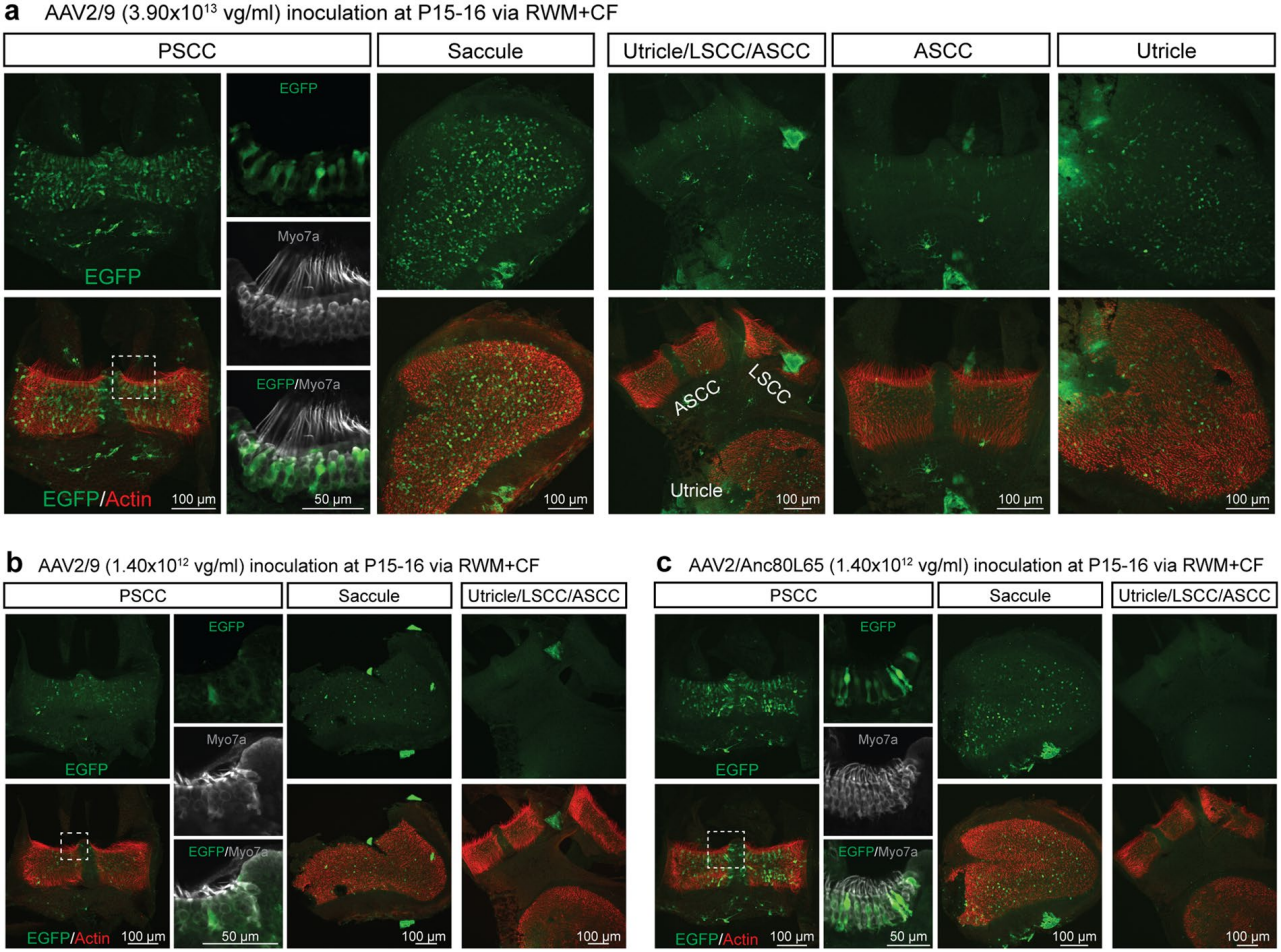
Vestibular organs are transduced following the RWM + CF injection. (**a**–**c**) Representative images of whole mounts of the CA of the PSCC, saccule, utricle and CAs of the LSCC and ASCC as indicated. Tissue was harvested 2 weeks after the RWM + CF injection (at P15–16), stained with Alexa Fluor 568-phalloidin (red) for labelling filamentous actin, and imaged for native eGFP (green). High magnification views of the regions marked with white dotted squares in the PSCC are shown separately and stained with Myo7a (gray) for labelling hair cells and imaged for native eGFP (green).

The vestibular organ is an equally important target for inner ear gene transfer. With AAV2/9 (3.90 × 10^13^ vg/ml), whole mounts of vestibular epithelia revealed robust eGFP expression in HCs of the crista ampullaris (CA) of the PSCC and in the saccule. We observed limited transduction of the sensory epithelia of the utricle and the CA of lateral semicircular canal (LSCC) and anterior semicircular canal (ASCC) (Fig. 5a). With low-titer AAV2/9 (1.40 × 10^12^ vg/ml), the transduction profile was similar, with an obvious dose-dependent effect (Fig. 5b). AAV2/Anc80L65 (1.40 × 10^12^ vg/ml) transduction was superior to that observed with low titer AAV2/9 (Fig. 5c).

**Figure 6:**
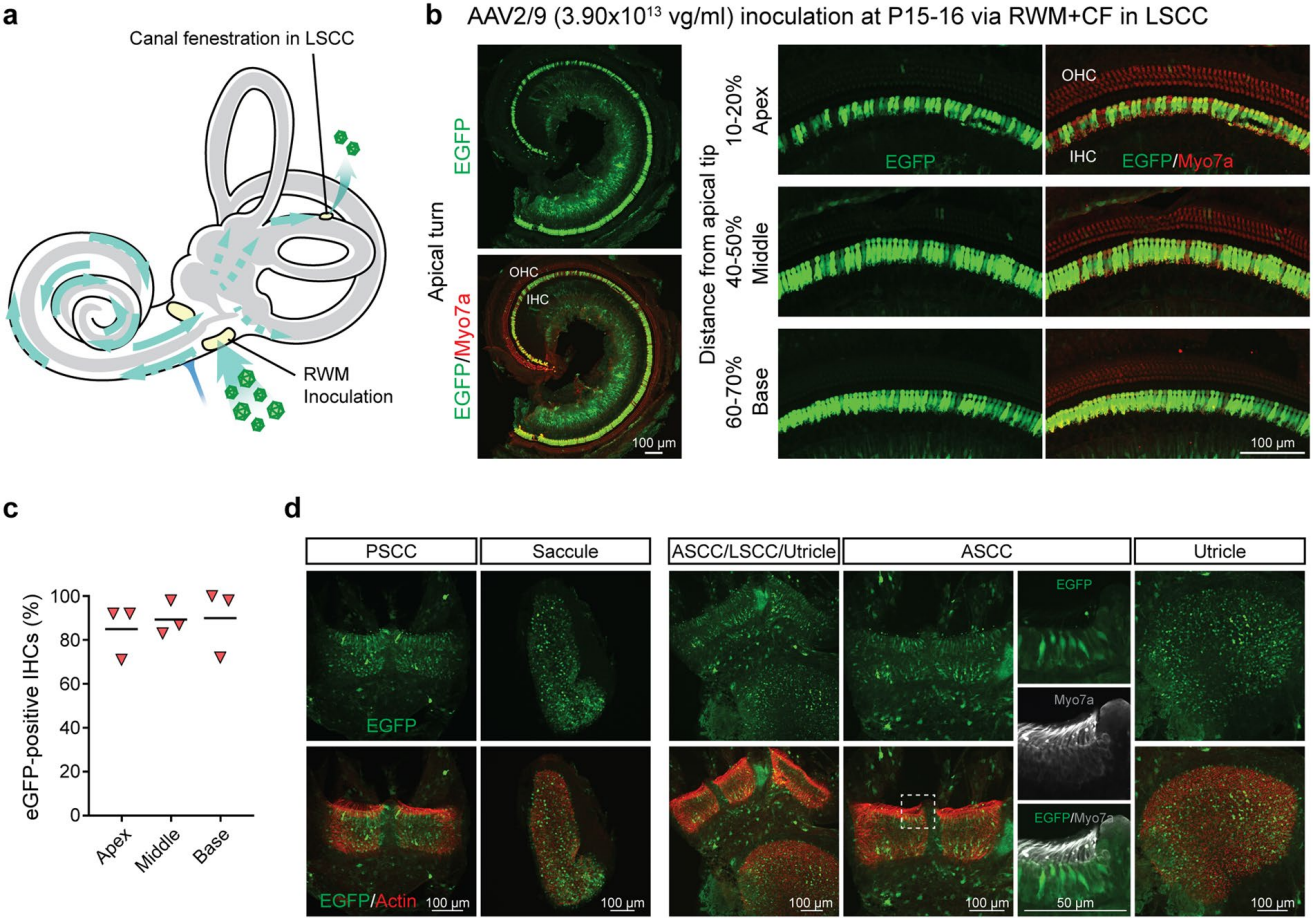
RWM + CF approach comparing canalostomy of the LSCC and PSCC. (**a**) Inner ear schematic showing the RWM + CF approach with a LSCC canalostomy. (**b**) Representative low magnification images of whole-mount apical turns and high magnification images of the apex, middle and base 2 weeks after injection of AAV2/9 (3.90 × 10^13^ vg/ml) delivered at P15–16 using a RWM + CF approach with a canalostomy in LSCC. Cochleae were stained with Myo7a (red) for labelling hair cells and imaged for native eGFP (green). (**c**) Quantification of eGFP-positive IHCs in the apex, middle and base, and cochlear apex 2 weeks following a RWM + CF approach with a canalostomy in LSCC (n = 3). Data are means. (**d**) Representative images of whole mounts of the CA of the PSCC, saccule, utricle and the CA of the LSCC and ASCC are shown. Tissue was harvested 2 weeks after injection of AAV2/9 (3.90 × 10^13^ vg/ml) delivered at P15–16 using a RWM + CF approach with a canalostomy in LSCC, and stained with Alexa Flour 568-phalloidin (red) for labelling filamentous actin and imaged for native eGFP (green). High magnification views of the regions marked with white dotted squares in the PSCC are shown separately, and stained with Myo7a (gray) for labelling hair cells and imaged for native eGFP (green).

We next compared fenestration of the PSCC and LSCC using high titer AAV2/9 (Fig. 6a). While both techniques resulted in similar transduction efficiency in cochlea (Fig. 6b and c), the inoculation pattern of the vestibular sensory epithelia was fenestration-site dependent. When the LSCC was fenestrated, eGFP expression was more pervasive and was observed throughout the vestibular sensory epithelia tissue (Fig. 6d). Irrespective of the canalostomy site, all mice had transient vestibular dysfunction as demonstrated by circling behavior after recovery from anesthesia, which resolved the following day.

### The cerebellum and contralateral ear: no transduction is seen

We examined whether the RWM or RWM + CF approaches resulted in off-target effects in the cerebellum or contralateral ear. Cross-sections of the cerebellum did not show any eGFP expression (Supplementary Fig. S2). We also observed no eGFP signal in the membranous labyrinth of the contralateral ear (Supplementary Fig. S2).

## Discussion

The next generation of hearing loss therapies will likely include therapeutic gene transfer to the cochlea. This treatment option requires the development of methods for safe, efficient delivery of transgene constructs to the relevant cell types in the organ of Corti. Amongst these cell types are the cochlear hair cells, which express approximately two-thirds of all genes implicated in non-syndromic sensorineural hearing loss (http://www.hereditaryhearingloss.org).

Transgene delivery to the inner ear is technically challenging because the target tissue, the organ of Corti, is exceptionally delicate and encased in a protective bony labyrinth. The latter limits the type of surgical approaches and when access is obtained, the gossamer-thin tissue of the membranous labyrinth makes unintended damage to the organ of Corti a significant possibility. Of surgical approaches used in murine models of hearing loss, access via the RWM has become the preferred route for gene delivery. The extent of transgene expression is dependent on the age of the animal at injection^2^, and while some investigators have demonstrated robust transduction of cells in the membranous labyrinth when injections are performed in adult mice, transduction is often limited to the basal turn of the cochlea or to scala tympani^11,12,17^. To address this limitation, direct delivery into the scala media has been explored, however this approach inevitably causes some cochlear damage, which leads to high frequency hearing loss^22^. Transduction efficiency is also highly variable^15,22^ and as a result, direct injection into the scala media is unlikely to become clinically applicable.

In this study, we focused on the mature cochlea. In initially testing the RWM approach, we found, like other investigators that IHC transduction was low and limited to the basal turn; in addition, the intervention itself caused mild auditory damage. Because transduction of apical cells is critical for gene therapies targeting the hearing loss in low frequencies^21^, we attempted an alternative delivery strategy – the RWM + CF approach. We reasoned that since the perilymphatic space is continuous between the cochlea and vestibule, a perilymph ‘exit hole’ in a semi-circular canal would reduce intracochlear viral-vector gradients and facilitate longitudinal flow of a viral vector throughout the cochlea.

Using the RWM + CF technique, we observed near complete transduction of cochlear IHCs with higher titers of AAV2/9 when we inoculated at P15–16. Delivery of higher titers of AAV2/9 at P56–60 also resulted in equally robust IHC transduction, suggesting that murine hair cell viral transduction does not decline with age, in contradistinction to observations reported in other studies^2,15^. We found no significant difference in auditory function between injected and uninjected ears at both P15–16 and P56–60. This finding is somewhat counterintuitive, as making a second fenestration could cause additional trauma. The fact that hearing thresholds are unaltered supports the need to vent inner ear fluid pressure when injecting so that pressure-induced damage does not occur.

We did find that a lower titer of AAV2/9 provided significantly less transduction of IHCs as compared to a higher titer, an observation consistent with other reports demonstrating that IHC transduction is dose dependent^10,20^. We also observed a serotype effect, which we demonstrated using AAV2/Anc80L65. This serotype showed significantly greater transduction efficiency when compared with equal titers of AAV2/9. However, with higher titers of AAV2/9, we also observed nearly 100% IHC transduction suggesting that both serotype and titer impact the amount of transduction that can be obtained^16,20^. We did observe that the number of transduced OHCs in the apex was lower than reported by other investigators using AAV2/Anc80L65 in adult mice^16^. This difference may reflect vector constructs, viral titers and/or inner ear injection route (endolymph vs. perilymph). Some reports utilizing AAV2/Anc80L65 vectors also carried Woodchuck Hepatitis Post-transcriptional Virus Regulatory Element (WPRE), which could increase eGFP expression levels^23^.

There are reports of attempts to restore auditory function in murine models of hearing loss by using AAV vectors to deliver therapeutics at later developmental or post-hearing time points. Using a RWM approach, Iizuka *et al*. delivered AAV5-*Gjb2* in an attempt to restore hearing in P42 *Gjb2* conditional knock-out mice^24^, and Pan *et al*. delivered AAV2/Anc80L65-*Ush1c* in an attempt to restore hearing in P10–12 Usher syndrome type 1c knock-in mice^7^. Neither of these experiments was successful in rescuing hearing in the treated animals, perhaps because the therapeutic window for correcting the hearing loss had closed or because transduction efficiency was too low.

The RWM + CF technique also transduces vestibular HCs. When a PSCC canalostomy is made, transduction of HCs in the CA of the PSCC is seen; in contrast, with a LSCC canalostomy, there is wide spread transduction of HCs in all vestibular organs. This difference suggests that the flow pattern created by the canalostomy is site dependent and may affect the transduction pattern within the vestibule.

In mice, the cochlear aqueduct provides a route for intercochlear communication, which can result in vector spread through the cerebrospinal fluid to the contralateral ear^25^. We hypothesized that the RWM + CF approach may limit off-target effects in the contralateral ear and cerebellum by providing a controlled egress for the inoculum. Consistent with this possibility, neither AAV serotype showed unwanted off-target effects with RWM + CF or RWM, suggesting that a 1μl injection volume is acceptable in mature mice and that leakage through the cochlear aqueduct is minimal to non-existent.

In summary, this study validates the RWM + CF approach as a safe technique to transduce cells of the organ of Corti in mature mice. Both AAV2/9 and AAV2/Anc80L65 enable widespread transduction of cochlear IHCs and vestibular HCs that is independent of the injection time point. We expect other conventional AAV serotypes and viral vectors to show similar enhanced transduction efficiency, albeit with tropism for different cochlear cell types. For example, we would predict that AAV1 will target OHCs and supporting cells, while AAV5 will transduce Claudius cells, spiral ganglion and inner sulcus cells^26^. These conventional AAVs have proven safety profile, however next generation AAVs like AAV2/Anc80L65 have not been evaluated for human trials and will likely be subject to careful scrutiny before wide-spread application^7^. Because the RWM + CF approach does not alter auditory function and does not have off target effects, its use will facilitate the completion of many of these studies focused on assessing the value of gene therapy in mature murine models of human deafness.

## Materials and Methods

### Ethics Approvals

All experiments were approved by the University of Iowa Institutional Biosafety Committee (IBC; rDNA Committee; RDNA Approval Notice #100024) and the University of Iowa Institutional Animal Care and Use Committee (IACUC; Protocol #06061787), and were performed in accordance with the NIH Guide for the Care and Use of Laboratory Animals.

### Virus Production

AAV2/Anc80L65 and AAV2/9 with a CMV-driven eGFP transgene cassette were prepared by the Gene Vector Core facility at the University of Iowa using the triple transfection method or baculovirus system, as described^27^. Viral titers were AAV2/9 at 3.90 × 10^13^ vg/ml 1.40 × 10^12^ vg/ml and AAV2/Anc80L65 at 1.40 × 10^12^ vg/ml. Virus aliquots were stored at −80 °C and thawed before use.

### Animal Surgery (RWM + CF injection, Supplementary Video S1)

Twenty-one wild-type C3HeB/FeJ mice (P15–16 and P56–60 of age) of both genders were used in this study. Mice were anesthetized with an intraperitoneal injection of ketamine (100 mg/kg) and xylazine (10 mg/kg). Body temperature was maintained with a heating pad during the surgical procedure. The left post-auricular region was shaved and cleaned.

Surgery was performed under an operating microscope. A post-auricular incision was made to access the temporal bone. The facial nerve was identified deeply along the wall of the external auditory canal (Fig. 1d). After exposing the facial nerve and the sternocleidomastoid muscle (SCM), a portion of the muscle was divided to expose the cochlea bulla ventral to the facial nerve. The posterior semicircular canal was exposed dorsal to the cochlea bulla (Fig. 1e).

A hole was gently drilled in the posterior semicircular canal with a 0.5 mm diameter diamond drill (ANSPACH^®^ EMAX2^®^ 2 Plus System, DePuy, Raynham, MA). Slow leakage of perilymph confirmed a patent canalostomy (Fig. 1f). While waiting 5–10 minutes for perilymph leakage to abate, a 0.5–1.0 mm diameter otologic drill was used to make a small hole in the cochlea bulla, which was then widened sufficiently with forceps to visualize the stapedial artery and the RWM (Fig. 1f). 1 μl of AAV with 2.5% fast green dye (Sigma-Aldrich, St. Louis, MO) was loaded into a borosilicate glass pipette (1.5 mm outer diameter [OD] × 0.86 mm inner diameter [ID], Harvard Apparatus, Hollison, MA) pulled with a Sutter P-97 micropipette puller to a final OD of ~20 µm and affixed to an automated injection system pressured by compressed gas (Harvard Apparatus). Pipettes were manually controlled with a micropipette manipulator.

The RWM was punctured gently in the center and AAV was microinjected into the scala tympani for 5 min (30–40 nL per injection) (Fig. 1g). Successful injections were confirmed by visualizing the efflux of green fluid from the PSCC canalostomy (Fig. 1g). After pulling out the pipette, the RWM niche was sealed quickly with a small plug of muscle to avoid leakage. The bony defect of the bulla and canal were then sealed with small plugs of muscles and Vetbond™ tissue adhesive (3 M, Maplewood, MN). 6–0 absorbable polypropylene sutures and 6–0 nylon monofilament sutures were used to close the SCM and skin, respectively.

Total surgical time ranged from 20–30 min for P15–16 mice and from 50–60 min for P56–60 mice. After all procedures, mice were placed on a heating pad for recovery and rubbed with bedding. P15–16 mice were returned to their mothers. Pain was controlled with buprenorphine (0.05 mg/kg) and flunixin meglumine (2.5 mg/kg) for 3 days. Recovery was closely monitored daily for at least 5 days post-operatively. All animals were operated on by one surgeon (HY). (Note: RWM inoculation was performed by excluding the canal fenestration procedure described above).

### Auditory Testing

ABRs were recorded as described previously^10^. All mice were anesthetized with an intraperitoneal injection of ketamine (100 mg/kg) and xylazine (10 mg/kg). All recordings were conducted from both ears of all animals on a heating pad, and electrodes were placed subcutaneously in the vertex, underneath the left or right ear. Clicks were square pulses 100 ms in duration and tone bursts were 3 ms in length at distinct 8, 16, 24 and 32 kHz frequencies. ABRs were measured with BioSigRZ (Tucker-Davis Technologies, Alachua, FL) for both clicks and tone bursts, adjusting the stimulus levels in 5-decibel (dB) increments between 25–90 dB sound pressure levels (SPL) in both ears. Electrical signals were averaged over 512 repetitions. ABR threshold was defined as the lowest sound level at which a reproducible waveform could be observed. ABRs were measured 2 weeks after the injection. Responses from the contralateral ear, which did not undergo surgery, were used as controls.

### Fluorescence Microscopy and Immunohistochemistry

Bilateral inner ears, brain and cerebellum were harvested 2 weeks after the intravenous injection. Deeply anesthetized animals were perfused transcardially with 4% paraformaldehyde for 15 min. Each tissue was locally perfused and fixed in 4% paraformaldehyde for 1 h at 4 °C, rinsed in PBS, and stored at 4 °C in preparation for immunohistochemistry. Specimens were visualized with a dissection microscope and dissected for whole-mount analysis. In all of the cochlear and vestibular whole mounts, native eGFP signals were observed. Following infiltration using 0.3% Triton X-100 for 30 min and blocking with 5% normal goat serum for 1 h, tissues were incubated with rabbit polyclonal Myosin-VIIA antibody (#25–6790, Proteus Biosciences Inc., Ramona, CA) diluted 1:200 in PBS for 1 h. For cochlear tissues, fluorescence-labeled goat anti-rabbit IgG Alexa Fluor 568 (#A-11036, Thermo Fisher Scientific, Rockford, IL) in 1:500 dilution was used as a secondary antibody for 30 min. For vestibular tissues, fluorescence-labeled goat anti-rabbit IgG Alexa Fluor 647 (#A-21244, Thermo Fisher Scientific) in 1:500 dilution was used as a secondary antibody for 30 min. Filamentous actin was labeled by a 30 min incubation of phalloidin conjugated to Alexa Fluor 568 (#A12380, Thermo Fisher Scientific) in 1:100 dilution. Specimens were mounted in ProLong™ Diamond Antifade Mountant with DAPI (#P36962, Thermo Fisher Scientific) and observed with a Leica TCS SP8 confocal microscope (Leica Microsystems Inc., Bannockburn, IL).

### Cell counts and transduction efficiency analysis

Cell counts and transduction efficiency analysis were performed as described previously^5,10^. Z stack images of whole mounts were collected at 10–20x on a Leica SP8 confocal microscope. Maximum intensity projections of z stacks were generated for each field of view, and images were prepared using LAS X (Leica Microsystems Inc.) to meet equal conditions. Inner hair cells with positive eGFP and overlapping Myo7a were counted per 400-μm sections per each turn for each specimen with ImageJ Cell Counter (NIH Image). The total number of HCs and GFP-positive HCs were summed and converted to a percentage. Any segments that contained dissection-related damage were omitted from the analysis.

### Statistical Analysis

Cell-counting and ABR data are presented as mean and mean ± S.E.M, respectively. Statistical analysis was performed using Prism 7 software package (GraphPad, San Diego, CA). Two groups were compared using unpaired two-tailed Student’s *t-*test. For comparisons of more than two groups, one-way ANOVA was performed and followed by post-hoc test with Bonferroni correction of pairwise group differences. *P* < 0.05 was considered statistically significant.

## Electronic supplementary material

Supplementary Information

Supplementary Video S1

## References

[1] Shibata SB, Raphael Y (2010). Future approaches for inner ear protection and repair. J. Commun. Disord..

[2] Akil O (2012). Restoration of hearing in the VGLUT3 knockout mouse using virally mediated gene therapy. Neuron.

[3] Lentz JJ (2013). Rescue of hearing and vestibular function by antisense oligonucleotides in a mouse model of human deafness. Nat. Med..

[4] Askew C (2015). Tmc gene therapy restores auditory function in deaf mice. Sci Transl Med.

[5] Shibata SB (2016). RNA Interference Prevents Autosomal-Dominant Hearing Loss. Am J Hum Genet.

[6] Isgrig K (2017). Gene Therapy Restores Balance and Auditory Functions in a Mouse Model of Usher Syndrome. Mol Ther.

[7] Pan B (2017). Gene therapy restores auditory and vestibular function in a mouse model of Usher syndrome type 1c. Nat. Biotechnol..

[8] Kelly MC, Chen P (2009). Development of form and function in the mammalian cochlea. Curr. Opin. Neurobiol..

[9] Muller U, Barr-Gillespie PG (2015). New treatment options for hearing loss. Nat Rev Drug Discov.

[10] Shibata SB, Yoshimura H, Ranum PT, Goodwin AT, Smith RJH (2017). Intravenous rAAV2/9 injection for murine cochlear gene delivery. Sci Rep.

[11] Liu Y (2005). Specific and efficient transduction of Cochlear inner hair cells with recombinant adeno-associated virus type 3 vector. Mol Ther.

[12] Chien WW, McDougald DS, Roy S, Fitzgerald TS, Cunningham LL (2015). Cochlear gene transfer mediated by adeno-associated virus: Comparison of two surgical approaches. Laryngoscope.

[13] Gantz, B. J. & Tyler, R. S. Cochlear implant comparisons. *Am*. *J*. *Otol*. ***Su****ppl*, 92–98 (1985). 3878092

[14] Fukui H, Raphael Y (2013). Gene therapy for the inner ear. Hear. Res..

[15] Shu Y (2016). Identification of Adeno-Associated Viral Vectors That Target Neonatal and Adult Mammalian Inner Ear Cell Subtypes. Hum. Gene Ther..

[16] Suzuki J, Hashimoto K, Xiao R, Vandenberghe LH, Liberman MC (2017). Cochlear gene therapy with ancestral AAV in adult mice: complete transduction of inner hair cells without cochlear dysfunction. Sci Rep.

[17] Staecker H, Li D, O’Malley BW, Van De Water TR (2001). Gene expression in the mammalian cochlea: a study of multiple vector systems. Acta Otolaryngol.

[18] Guo JY (2017). Cochleovestibular gene transfer in neonatal mice by canalostomy. Neuroreport.

[19] Kawamoto K, Oh SH, Kanzaki S, Brown N, Raphael Y (2001). The functional and structural outcome of inner ear gene transfer via the vestibular and cochlear fluids in mice. Mol Ther.

[20] Landegger LD (2017). A synthetic AAV vector enables safe and efficient gene transfer to the mammalian inner ear. Nat. Biotechnol..

[21] Borkholder DA (2010). Murine intracochlear drug delivery: reducing concentration gradients within the cochlea. Hear. Res..

[22] Kilpatrick LA (2011). Adeno-associated virus-mediated gene delivery into the scala media of the normal and deafened adult mouse ear. Gene Ther..

[23] Loeb JE, Cordier WS, Harris ME, Weitzman MD, Hope TJ (1999). Enhanced expression of transgenes from adeno-associated virus vectors with the woodchuck hepatitis virus posttranscriptional regulatory element: implications for gene therapy. Hum. Gene Ther..

[24] Iizuka T (2015). Perinatal Gjb2 gene transfer rescues hearing in a mouse model of hereditary deafness. Hum. Mol. Genet..

[25] Stover T, Yagi M, Raphael Y (2000). Transduction of the contralateral ear after adenovirus-mediated cochlear gene transfer. Gene Ther..

[26] Ahmed H, Shubina-Oleinik O, Holt JR (2017). Emerging Gene Therapies for Genetic Hearing Loss. J Assoc Res Otolaryngol.

[27] Yang GS (2002). Virus-mediated transduction of murine retina with adeno-associated virus: effects of viral capsid and genome size. J. Virol..

