# Supplementary material for: Enhanced viral-mediated cochlear gene delivery in adult mice by combining canal fenestration with round window membrane inoculation
Source: Sci Rep. 2018 Feb 14;8:2980. doi: 10.1038/s41598-018-21233-z (PMC5812997; doi:10.1038/s41598-018-21233-z)
Supplement: Supplementary file 1 — Supplementary Information [file 41598_2018_21233_MOESM1_ESM.pdf]

Supplementary Information

Enhanced viral-mediated cochlear gene delivery in adult mice by combining canal fenestration with round window membrane inoculation

Hidekane Yoshimura [1,2†], Seiji B. Shibata [1,3†,] Paul T. Ranum[1,4]
and Richard J.H. Smith[1,3,4,5*]

[1] Molecular Otolaryngology and Renal Research Laboratories, Carver College of Medicine, University of Iowa, Iowa City, IA 52242, USA

[2] Department of Otorhinolaryngology, Shinshu University School of Medicine, Matsumoto, Nagano 390-8621, Japan

[3] Department of Otolaryngology - Head and Neck Surgery, Carver College of Medicine, University of Iowa, Iowa City, IA 52242, USA

[4] Interdisciplinary Graduate Program in Molecular & Cellular Biology, The University of Iowa Graduate College, University of Iowa, Iowa City, IA 52242, USA

[5] Iowa Institute of Human Genetics, Carver College of Medicine, University of Iowa, Iowa City, IA 52242, USA

[†] These authors contributed equally

Supplementary Figure Legends

Supplementary Figure S1: Comparative auditory function following RWM and RWM+CF approaches to the inner ear in adult mice. Representative click ABR tracings (70-35 dB SPL) recorded from injected and uninjected ears 2 weeks after inoculation shows mild auditory damage associated with the RWM approach.

Supplementary Figure S2: Neither the RWM nor RWM+CF approach in adult mice affects the cerebellum and contralateral ear. Representative single-channel and merged cross-sectional images of the cerebellum stained with anti-GFP (green) antibody and DAPI for labelling nuclei and whole-mount images of the uninjected contralateral ears stained with Myo7a (red) for labelling hair cells and imaged for native eGFP (green) as indicated. Scale bar: 100 µm.

Supplementary Video S1: Representative surgical movies showing the RWM+CF injection. Surgery was performed on P15-16 mice under a surgical microscope (M220 F12, Leica Microsystems, Germany) (magnification, 16-40x; SCM, sternocleidomastoid muscle; LSCC, lateral semicircular canal; PSCC, posterior semicircular canal; RWM, round window membrane).

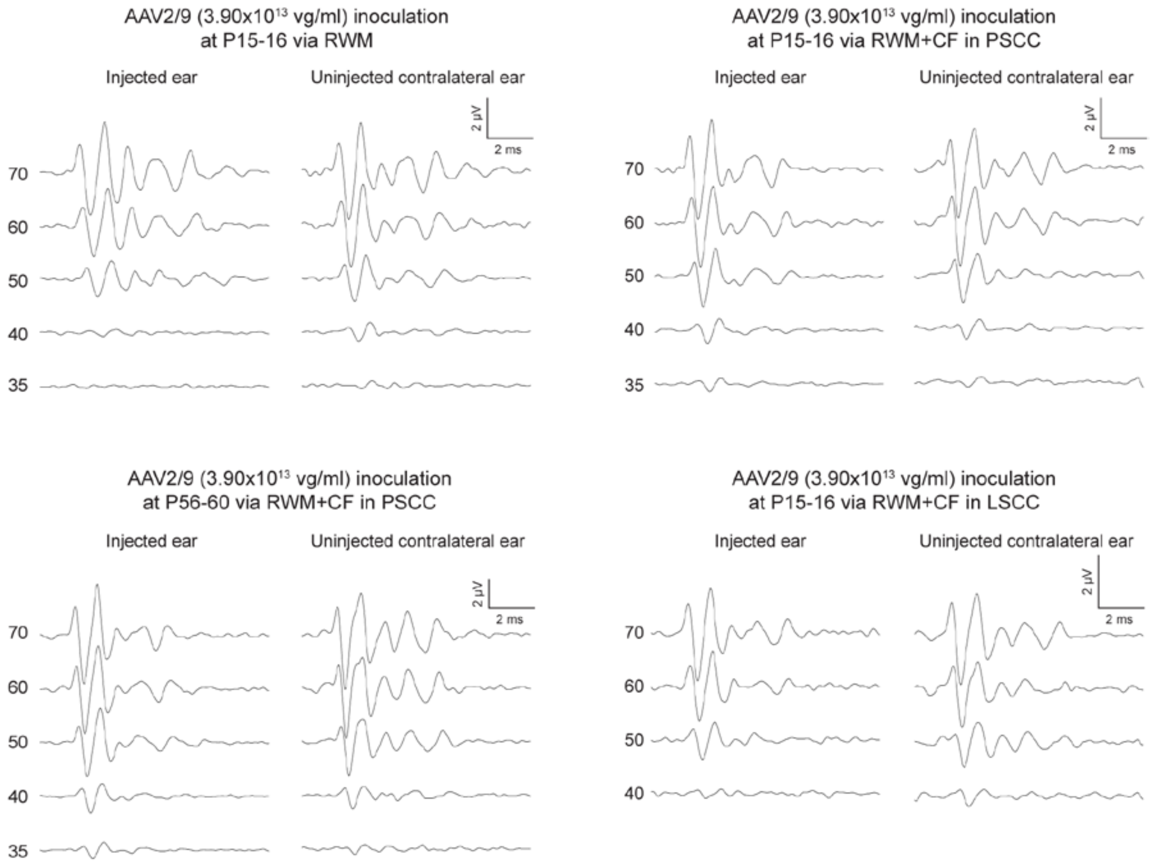
AAV2/9 (3.90x10[13] vg/ml) inoculation
at P15-16 via RWM
Injected ear
Uninjected contralateral ear
2 µV
2 ms
70
60
50
40
35
AAV2/9 (3.90x10[13] vg/ml) inoculation
at P15-16 via RWM+CF in PSCC
Injected ear
Uninjected contralateral ear
AAV2/9 (3.90x10[13] vg/ml) inoculation
at P56-60 via RWM+CF in PSCC
Injected ear
Uninjected contralateral ear
AAV2/9 (3.90x10[13] vg/ml) inoculation
at P15-16 via RWM+CF in LSCC
Injected ear
Uninjected contralateral ear

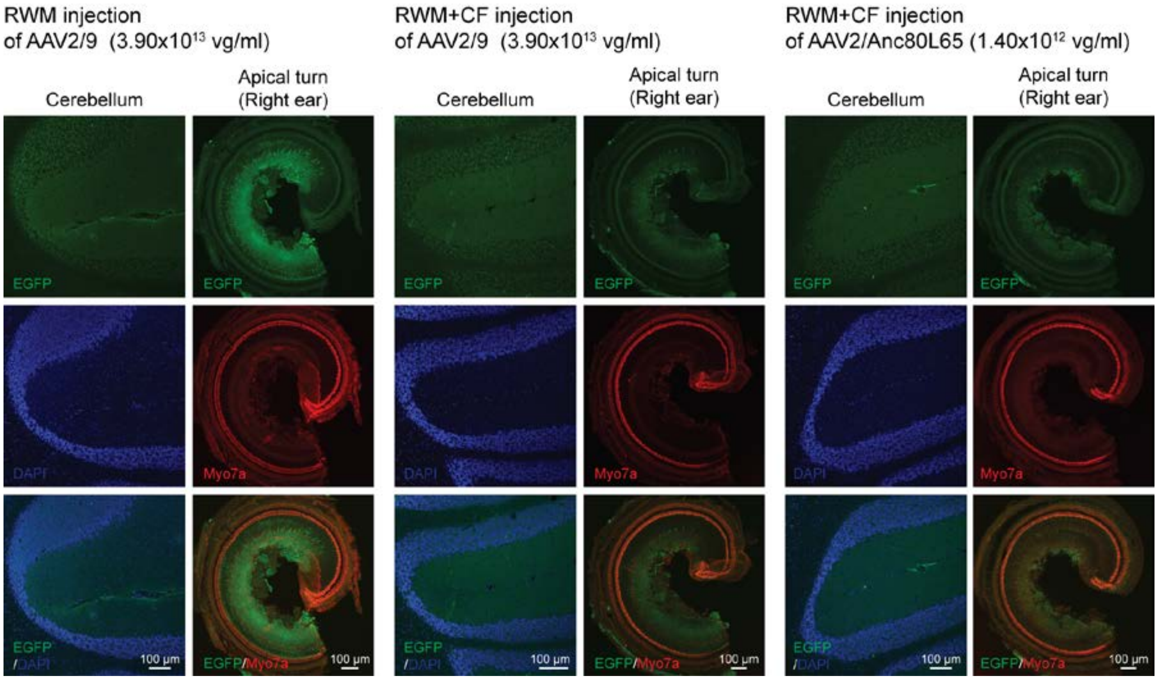
RWM injection
of AAV2/9 (3.90x10^13 vg/ml)
RWM+CF injection
of AAV2/9 (3.90x10^13 vg/ml)
RWM+CF injection
of AAV2/Anc80L65 (1.40x10^12 vg/ml)
Cerebellum
Apical turn
(Right ear)
EGFP
DAPI
Myo7a
EGFP/DAPI
EGFP/Myo7a
100 µm
